# Scoring amino acid mutation to predict pandemic risk of avian influenza virus

**DOI:** 10.1186/s12859-019-2770-0

**Published:** 2019-06-10

**Authors:** Xiaoli Qiang, Zheng Kou

**Affiliations:** Institute of Computing Science and Technology, Guangzhou University, 230 Wai Huan Xi Road, Guangzhou Higher Education Mega Center, Guangzhou, 510006 People’s Republic of China

**Keywords:** Avian influenza virus, Amino acid mutation, Machine learning, Pandemic risk

## Abstract

**Background:**

Avian influenza virus can directly cross species barriers and infect humans with high fatality. As antigen novelty for human host, the public health is being challenged seriously. The pandemic risk of avian influenza viruses should be analyzed and a prediction model should be constructed for virology applications.

**Results:**

The 178 signature positions in 11 viral proteins were firstly screened as features by the scores of five amino acid factors and their random forest rankings. The Supporting Vector Machine algorithm achieved well performance. The most important amino acid factor (Factor 5) and the minimal range of signature positions (63 amino acid residues) were also explored. Moreover, human-origin avian influenza viruses with three or four genome segments from human virus had pandemic risk with high probability.

**Conclusion:**

Using machine learning methods, the present paper scores the amino acid mutations and predicts pandemic risk with well performance. Although long evolution distances between avian and human viruses suggest that avian influenza virus in nature still need time to fix among human host, it should be notable that there are high pandemic risks for H7N9 and H9N2 avian viruses.

**Electronic supplementary material:**

The online version of this article (10.1186/s12859-019-2770-0) contains supplementary material, which is available to authorized users.

## Background

Influenza A virus contains eight segments of single-strand negative RNA. Segment 4 codes hemagglutinin (HA) gene and segment 6 codes neuraminidase (NA) gene. According to the antigenic characteristics of HA and NA, avian influenza A virus has 16 subtypes HA and nine subtypes NA [1]. Since the mutation rates of viral genome were fast, the phenotype of antigen, drug-resistance, and virulence changed in a relative short time. Moreover, segmental pattern facilitates the reassortment of viral genome and promote fast change of phenotypes [1].

Avian influenza virus (AIV) could across the species barrier and infect human fatally, which caused huge loss of economy and attracted extensive attention of the society. The highly pathogenic AIV of H5N1 subtype was firstly reported in Asia in 1996 [2]. The fact that H5N1 virus cross species barriers directly and fatally infect the respiratory system were confirmed by the isolation of human-origin H5N1 virus from clinical samples in 1997 [3, 4]. Human infections of H5N1 subtype were continuously reported widely since 2003 and huge data were deposited in public database [5–8]. Besides H5N1 virus, other subtypes can also infect human by direct interspecies transmission. There are two infection cases of H9N2 in 1999 and 2003 [9, 10]. H7N7 virus infected farmers in the Netherlands in 2003 [11], Moreover, H7N9 occurred in 2013 and infections of human cases were still reported up to now [12, 13]. Interspecies transmission of AIV had two phenotypes in the view of transmission efficiency: (1) keeping popular among poultry or causing human infection with low probability; (2) adaptation to human host and human-to-human transmission with high efficiency. Thus far, AIVs in nature had not the second phenotype, which represents initial adaption to the new host and low efficiency of transmission among human.

Seasonal and pandemic influenza virus had high efficiency of transmission among human. Unfortunately, more and more reports about transmission efficiency proved that AIV with adequate amino acid (AA) mutations could have the ability of highly efficient transmission among mammals, which strongly suggested that pandemic risk of AIVs among human was rising [14–20]. As high fatality and antigen novelty for human host, the public health is being challenged seriously by AIVs. So, computational tools in the field of bioinformatics should be proposed to screen mutations in viral proteins not only for the study of high efficiency transmission among human but also for the prediction of transmission phenotype and the corresponding pandemic risk of AIVs.

In a previous study, five amino acid factors summarized from 491 highly redundant amino acid attributes were associated with specific physiochemical amino acid properties, namely, polarity, secondary structure, molecular volume, codon diversity, and electrostatic charge [21]. In this paper, we used five AA factors to transform viral proteins and used the random forest (RF) method to select features from high-dimensional protein data and score them by their contributions to the efficiency of transmission and pandemic risk. After ranking the positions containing important mutation information, the classifier could predict the transmission phenotype of high efficiency to evaluate the pandemic risk. In the paper, we first identified 178 signature mutation positions by the RF scoring, then predicted AIV occurrence by four popular machine learning methods. Using the most effective classifier, we explored the important amino acid factors and the minimal range of signature positions. The study results could benefit pandemic surveillance and future study on the efficiency of AIV transmission.

## Results

### Dataset

The final dataset contained 869 high-quality AIV strains (440 avian-origin AIVs with H1–H14, H16 subtypes; 429 human-origin AIVs with H5N1, H5N6, H7N3, H7N7, H7N9 and H9N2 subtypes) and 914 seasonal, pandemic human, and artificial viruses (H1N1, H1N2, H3N2 subtype; H5N1 artificial virus). As the 869 AIVs have low efficiency of transmission and low pandemic risk among human, they were regarded as negative samples. The 914 human or artificial viruses were regarded as positive samples since they were verified to have high efficiency of transmission among humans or mammals. The information related to these strains is summarized in Additional file 1.

### Signature amino acid residues

**Fig. 1:**
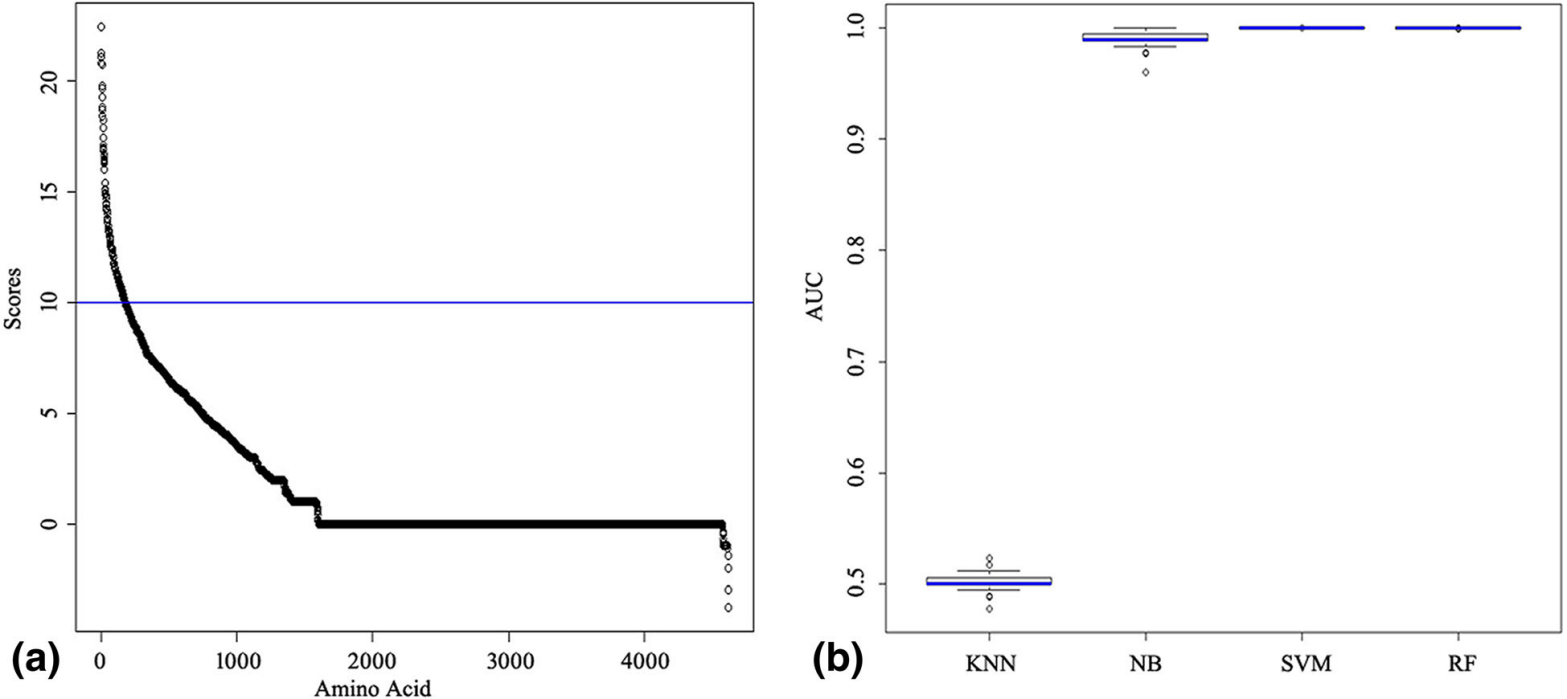
Importance score curve and the performances of k-nearest neighbor (KNN), naïve Bayes (NB), support vector machine (SVM), and random forest (RF) classifiers. **a** The ranked scores were calculated from five AA factors using the random forest method. The x and y coordinates denote the total length of the 11 protein alignments and the importance scores, respectively. The cutoff value 10 is indicated by the thin horizontal line. **b** Performances of the four classifiers were evaluated from 100 repeats of 10-fold cross-validation. The area under the curve (AUC) ranges from 0 to 1

The importance score at each position in the 11 viral proteins was computed by the RF model to screening the signature positions. The slope of the curve obviously changed at an importance score of 10 (Fig. 1a). Therefore, 10 was preliminary selected as cutoff score. The 178 signature positions were founded and the initial amino acid mutation set was generated for further machine learning.

As shown in Table 1, the hemagglutinin protein (HA) contained the largest number of signature positions (41 amino acid residues; about 41/178 = 23%), suggesting that HA is very important for highly efficient transmission of AIVs among human. HA is mainly involved in receptor-binding and fusion activities. Positions HA102-HA290 locate in or close to the region of host receptor binding [22, 23], and HA158, H163, HA189, HA190, HA224, HA226, HA228H is reportedly related to the specificity of receptor binding [14–19]. HA94, HA101, HA327, HA367, and HA393 locate at or near the fusion peptide [24], which triggers fusion activity in acidic environments and favors transmission to humans. The HA327 position in the cleavage site are important virulence sites [25]. The 627 position in the polymerase basic protein 2 (PB2) has been implicated in increased replication or virulence of AIVs in mammals and transmission among humans [19, 26]. The 93 and 95 positions in the matrix protein 2 (M2), which are affiliated with viral particle ensembles [27], were also screened. The 372 and 375 positions in the nucleoprotein (NP) are reportedly involved in intracellular transport of viral proteins [28, 29].

**Table 1:** Scores for the 178 signature amino acids of avian influenza viruses

Num	Pro^a^	Pos^b^	Score	Num	Pro	Pos	Score	Num	Pro	Pos	Score
1	PB2	44	12.13	61	HA	124	12.51	121	NP	430	10.74
2	PB2	61	14.04	62	HA	137	10.16	122	NP	442	18.71
3	PB2	81	11.32	63	HA	141	10.61	123	NP	444	12.48
4	PB2	105	13.20	64	HA	144	10.61	124	NP	455	10.90
5	PB2	199	12.53	65	HA	155	10.56	125	NP	456	10.54
6	PB2	225	10.72	66	HA	158	10.36	126	NP	473	10.14
7	PB2	271	21.13	67	HA	160	16.89	127	NA	105	10.92
8	PB2	323	20.77	68	HA	163	11.46	128	NA	200	10.32
9	PB2	368	10.29	69	HA	164	10.57	129	NA	247	17.48
10	PB2	391	13.25	70	HA	169	10.33	130	NA	347	10.16
11	PB2	475	16.03	71	HA	171	10.35	131	NA	372	10.85
12	PB2	526	11.05	72	HA	172	10.15	132	NA	399	12.30
13	PB2	559	10.52	73	HA	189	13.15	133	M1	15	10.57
14	PB2	567	12.55	74	HA	190	19.80	134	M1	30	16.61
15	PB2	588	14.51	75	HA	193	13.31	135	M1	37	10.92
16	PB2	591	10.79	76	HA	203	12.47	136	M1	115	16.49
17	PB2	627	11.12	77	HA	224	16.94	137	M1	116	19.25
18	PB2	645	11.76	78	HA	225	14.94	138	M1	137	11.12
19	PB2	674	11.02	79	HA	226	15.14	139	M1	142	11.79
20	PB1	99	16.40	80	HA	228	15.09	140	M1	207	12.04
21	PB1	287	11.47	81	HA	246	10.95	141	M1	209	14.74
22	PB1	336	14.27	82	HA	272	12.19	142	M1	214	16.99
23	PB1	339	11.25	83	HA	276	12.12	143	M2	13	11.26
24	PB1	361	13.80	84	HA	285	10.01	144	M2	14	12.88
25	PB1	368	12.23	85	HA	299	10.21	145	M2	18	12.14
26	PB1	375	13.66	86	HA	327	12.71	146	M2	20	10.14
27	PB1	486	11.37	87	HA	367	11.62	147	M2	27	14.13
28	PB1	581	19.70	88	HA	393	13.32	148	M2	28	10.01
29	PB1	584	11.53	89	HA	406	11.53	149	M2	31	11.00
30	PB1	741	14.47	90	HA	413	10.90	150	M2	43	13.76
31	PB1_f2	11	11.53	91	HA	462	11.17	151	M2	50	10.52
32	PB1_f2	27	13.74	92	HA	490	10.82	152	M2	54	12.38
33	PB1_f2	59	10.05	93	HA	493	11.39	153	M2	57	11.57
34	PB1_f2	60	14.89	94	HA	530	10.65	154	M2	65	11.31
35	PB1_f2	73	11.33	95	HA	531	12.94	155	M2	66	13.29
36	PB1_f2	78	13.81	96	NP	16	11.77	156	M2	77	10.00
37	PB1_f2	83	14.22	97	NP	21	10.30	157	M2	78	17.00
38	PA	28	16.42	98	NP	33	15.12	158	M2	79	12.74
39	PA	55	13.03	99	NP	61	12.44	159	M2	86	15.40
40	PA	57	11.25	100	NP	99	22.49	160	M2	93	18.45
41	PA	65	10.78	101	NP	100	18.84	161	M2	95	11.66
42	PA	66	10.42	102	NP	119	10.82	162	NS1	7	10.23
43	PA	94	13.02	103	NP	136	13.19	163	NS1	22	11.57
44	PA	163	11.54	104	NP	189	11.58	164	NS1	53	10.59
45	PA	225	12.94	105	NP	190	13.23	165	NS1	60	13.19
46	PA	268	12.08	106	NP	283	17.10	166	NS1	74	10.03
47	PA	277	14.17	107	NP	289	11.37	167	NS1	81	14.88
48	PA	337	13.50	108	NP	293	12.50	168	NS1	114	13.45
49	PA	391	10.69	109	NP	305	20.80	169	NS1	125	11.55
50	PA	400	11.18	110	NP	313	16.72	170	NS1	171	10.83
51	PA	421	12.85	111	NP	345	13.40	171	NS1	189	13.00
52	PA	520	11.20	112	NP	351	10.14	172	NS1	205	11.00
53	PA	552	16.33	113	NP	353	10.09	173	NS1	215	11.37
54	PA	669	11.64	114	NP	357	21.26	174	NS1	227	12.45
55	HA	12	14.87	115	NP	372	12.50	175	NEP	32	12.43
56	HA	94	10.48	116	NP	375	12.09	176	NEP	70	18.26
57	HA	101	11.00	117	NP	400	10.68	177	NEP	89	11.59
58	HA	110	17.94	118	NP	422	14.84	178	NEP	107	14.74
59	HA	111	11.00	119	NP	425	12.20				
60	HA	117	11.34	120	NP	426	10.69				

^a^Viral protein; ^b^Position of amino acid residue as H3 subtype numbering

The viral proteins were transformed by the five amino acid factors and 178 signature positions were screened by the RF method. Part of the signature positions had been verified to be related with the mechanism of interspecies transmission or high efficiency of transmission among humans, which would rationalize model construction and benefit predicting accuracy. Moreover, the rest amino acid mutation without trial verification would facilitate the exploration of molecular mechanisms about high efficiency transmission among humans.

### Performance of the prediction model

The 10-fold cross validation and the receiver operating characteristic (ROC) curve were used to evaluate the performance of the classifiers. The area under the ROC curve (AUC) reveals the optimal parameters in the four classifiers. As shown in Fig. 1b, the performances were different obviously. The AUC medians of the Supporting Vector Machine (SVM) and RF models were almost 1 while that for the K-Nearest Neighbor (KNN) model were almost 0.5. The KNN model had not good performance and the reason may be the nonlinear prediction rules in feature space. The performance of the Naïve Bayes (NB) classifier was slightly poorer and less stable than those of the SVM and RF classifiers. Considering the benefit of small samples and the computation complex, the SVM classifier was selected as the optimal machine learning model for predicting pandemic risk of AIVs.

### Contributions of the AA factors

**Fig. 2:**
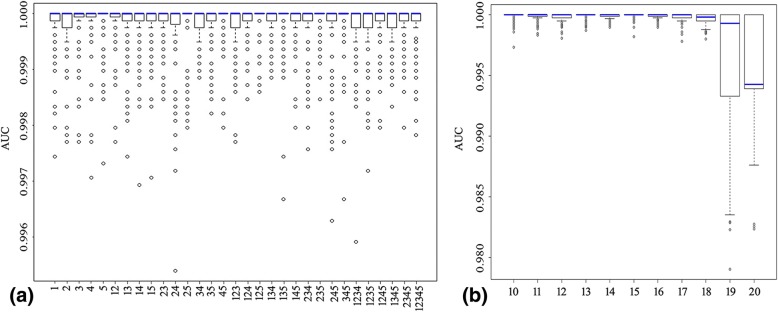
Contributions of AA factors and different mutation sets. **a** Performance of SVM classifier for different combinations of the five AA factors. The *x* and *y* coordinates denote the 31 combination patterns and the AUC values (from 0 to 1), respectively. Along the x axis, ‘15’ denotes that the set of 178 amino acid residues was transformed using AA Factor 1 and AA Factor 5 together, for example. **b** Contributions of mutation positions for different cutoff values (range 10–20). The *y* coordinate shows the AUC values

AIVs were characterized by the scores of 178 amino acid mutations. The five AA factors were associated with specific physiochemical amino acid properties: polarity, secondary structure, molecular volume, codon diversity, and electrostatic charge. To understand the importance of the five AA factors, the SVM classifier was used to evaluate all combination patterns. As shown in Fig. 2a, most of the stable performances of the SVM classifier were contributed by AA Factor 5 or combinations with AA Factor 5. Notably, the median AUC values were almost 1 and remained stable under AA Factor 5 alone. The performances of the SVM classifiers under AA Factor 1, or AA Factor 2 alone were not as good as AA Factor 5. These results indicate an important role for AA Factor 5 in the mechanism of AIVs transmission. Therefore, AA Factor 5 was employed in further analysis.

### Contributions of the mutation sets

**Fig. 3:**
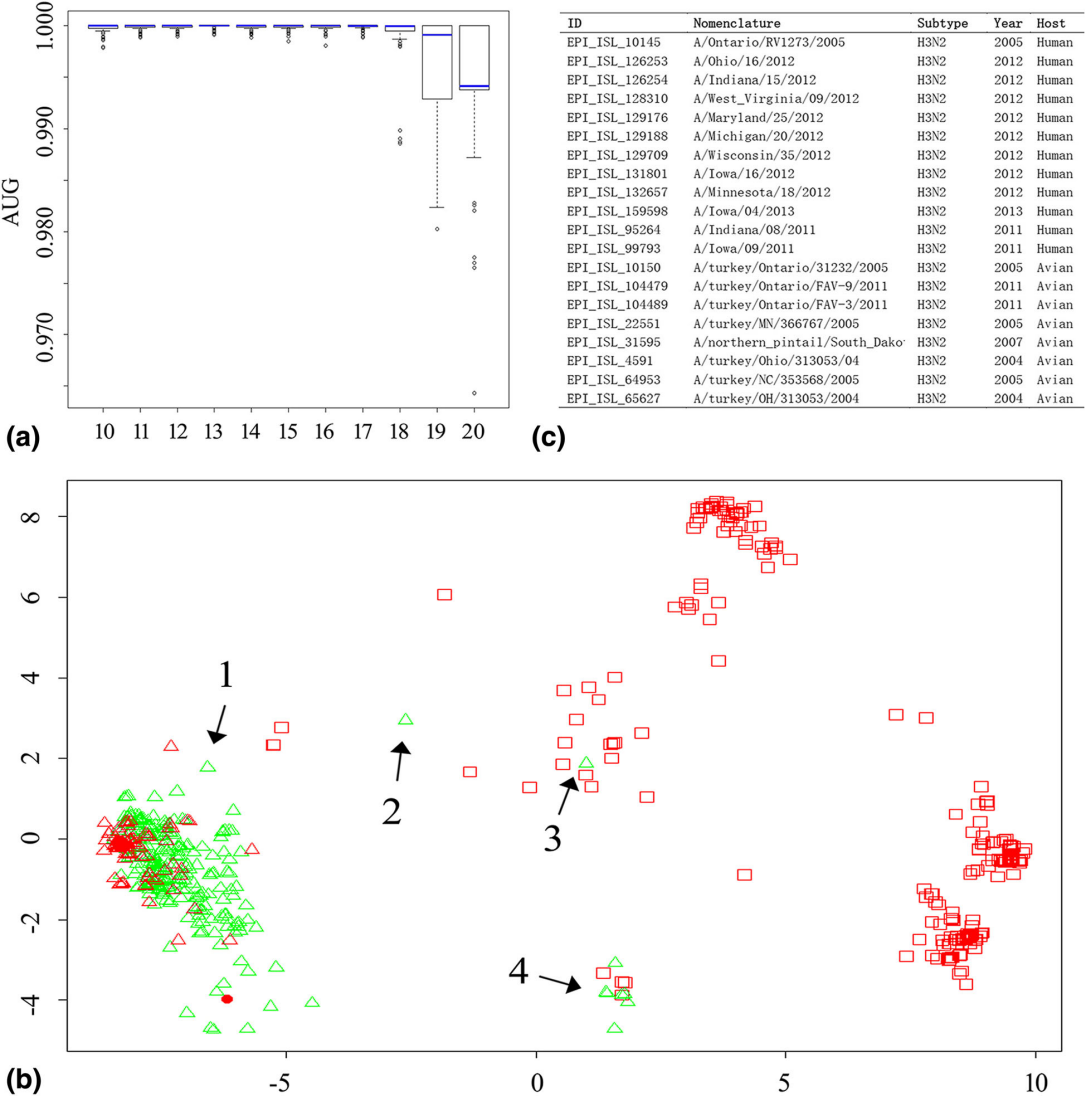
The distribution of influenza viruses by minimal amino acid set. **a** Contributions of reduced mutation position sets. The *x* and *y* coordinates denote the cutoff (range 10–20) and the AUC values (range 0–1), respectively. **b** Patterns of human and avian influenza viruses clustered by the multidimensional scaling (MDS) method. Avian influenza viruses were marked by hollow triangle (Red, human-origin; Green, avian-origin). Seasonal and pandemic human influenza viruses were marked by red hollow rectangle. The six artificial H5N1 virus were marked by red solid circle. **c** human and avian influenza H3N2 viruses in group 4

One hundred seventy-eight mutation sites were achieved under a cutoff value of 10 as mentioned above. To further explore the minimum mutations set associated with transmission efficiency, the cutoff value was adjusted and was incremented in steps of 1. The SVM classifier was still calculated with the five AA factors together. As shown in Fig. 2b, the SVM classifier destabilized at higher cutoffs and achieved stable and best performance at cutoffs 13. The performance of the SVM classifier with AA Factor 5 alone was also calculated for different cutoffs. As shown in Fig. 3a, the SVM classifier performed stably and well up to a cutoff of 17 and the best performance was achieved at cutoff 13, which giving 63 signature positions (Table 2). These 63 signature residues were regarded as the minimum mutation set of amino acid residues and were transformed by AA Factor 5 alone to show the pattern of avian and human influenza viruses by the multidimensional scaling method [see Additional file 2].

**Table 2:** Minimal amino acid set for predicting AIVs

Num	Pro^a^	Pos^b^	Score	Num	Pro	Pos	Score	Num	Pro	Pos	Score
1	PB2	61	14.04	22	PA	337	13.50	43	NP	345	13.40
2	PB2	105	13.20	23	PA	552	16.33	44	NP	357	21.26
3	PB2	271	21.13	24	HA	12	14.87	45	NP	422	14.84
4	PB2	323	20.77	25	HA	110	17.94	46	NP	442	18.71
5	PB2	391	13.25	26	HA	160	16.89	47	NA	247	17.48
6	PB2	475	16.03	27	HA	189	13.15	48	M1	30	16.61
7	PB2	588	14.51	28	HA	190	19.80	49	M1	115	16.49
8	PB1	99	16.40	29	HA	193	13.31	50	M1	116	19.25
9	PB1	336	14.27	30	HA	224	16.94	51	M1	209	14.74
10	PB1	361	13.80	31	HA	225	14.94	52	M1	214	16.99
11	PB1	375	13.66	32	HA	226	15.14	53	M2	27	14.13
12	PB1	581	19.70	33	HA	228	15.09	54	M2	43	13.76
13	PB1	741	14.47	34	HA	393	13.32	55	M2	66	13.29
14	PB1_f2	27	13.74	35	NP	33	15.12	56	M2	78	17.00
15	PB1_f2	60	14.89	36	NP	99	22.49	57	M2	86	15.40
16	PB1_f2	78	13.81	37	NP	100	18.84	58	M2	93	18.45
17	PB1_f2	83	14.22	38	NP	136	13.19	59	NS1	60	13.19
18	PA	28	16.42	39	NP	190	13.23	60	NS1	81	14.88
19	PA	55	13.03	40	NP	283	17.10	61	NS1	114	13.45
20	PA	94	13.02	41	NP	305	20.80	62	NEP	70	18.26
21	PA	277	14.17	42	NP	313	16.72	63	NEP	107	14.74

^a^Viral protein; ^b^Position of amino acid residue as H3 subtype numbering

The distribution of human and avian influenza virus in two dimensions were shown in Fig. 3b. In the view of pandemic risk, most of avian viruses were cluster at the low left while human viruses formed three separate clusters at the right. Avian influenza virus 1 (EPI_ISL_64953, A/turkey/NC/353568/2005, H3N2), 2 (EPI_ISL_3141, A/Duck/Nanchang/4–184/2000, H2N9) and 3 (EPI_ISL_3362, A/duck/NC/91347/2001, H1N2) were closed to the human viruses, which should be strictly supervised in the future. The viruses in group 4 were composed by seasonal human and avian virus of H3N2 subtype isolated from 2005 to 2013 in North America (Fig. 3c), which suggested that direct interspecies transmission once occurred.

As shown in Table 2, the 63 signature positions were screened with the cut-off value 13. The nucleoprotein (NP) contained the largest number of signature positions (12 amino acid residues; about 12/63 = 19%), suggesting that NP is very important for host range of influenza virus [1]. The HA protein contained the similar number of signature positions to the NP protein (11 amino acid residues; about 11/63 = 17%), which further confirmed that HA is very important for highly efficient transmission of AIVs among human. Although amino acid mutations in the HA protein are essential for AIV transmission in mammals [14–19], mutations in other proteins are also necessary and should be further verified by trials [14, 15, 20]. Mutations distribution in different viral proteins suggested that the role of synergy and nonlinearity among viral proteins should be focused in the study of AIVs.

### Pandemic risk of human-origin AIVs

It was supposed that potential pandemic may be triggered by the reassortment of viral genomes [1], which means that genome segments of human viruses (excluding the HA segment) were inserted into the genome of AIVs. To value the pandemic risk of human-origin AIVs, the artificial stimulation of genome reassortment between human-origin AIVs and human influenza viruses (seasonal human virus and 2009 pandemic virus) was performed. As shown in Table 3, three or four genome segments were needed at least to achieve the change of transmission phenotype with high probability (> = 0.90). The computing results were compatible with the reports from Zhang Y., et al. 2013 [20]. It should be notable that there was high pandemic risk for H7N9 virus (only three segments needed) and H9N2 virus (flexible patterns of genome reassortment), which was very important for the surveillance of avian influenza virus in the future.

**Table 3:** Artificial simulation of genome reassortment

Human-origin AIV	Human influenza virus	Probability	Genome segment
A/Egypt/682/2015_H5N1	A/Ohio/09/2015_H1N1	0.90	seg1seg3seg5seg7
A/Zhejiang/9/2015_H7N9	A/Ohio/09/2015_H1N1	0.91	seg1seg5seg7
A/Hunan/44558/2015_H9N2	A/Ohio/09/2015_H1N1	0.94	seg1seg2seg5seg7
A/Hunan/44558/2015_H9N2	A/Ohio/09/2015_H1N1	0.93	seg1seg3seg5seg7
A/Hunan/44558/2015_H9N2	A/Ohio/09/2015_H1N1	0.91	seg1seg5seg7seg8
A/Hunan/44558/2015_H9N2	A/Sichuan/1/2009_H1N1	0.91	seg1seg2seg5seg7

## Discussion

Avian influenza viruses can cross the species barrier, potentially causing a human pandemic. In this paper, AIV pandemic risk was predicted by the SVM model with excellent performance. We firstly screened 178 mutation positions in the 11 viral proteins by the RF method. Part of the residues at these positions have been related to interspecies transmission in earlier reports, such as HA158, H163, HA189, HA190, HA224, HA226, HA228H [14–16, 18], H163 [17], HA94, HA101, HA327, HA367, and HA393 [24], M2 93, M2 95 [27], NP372, NP375 [28, 29], PB2 627 [26], which guarantee the accuracy and the biologically meaningful of the predicting model. The proposed models provide important clues for future surveillance in the field of virology and is a useful pre-screening tool for phenotype screening in high-level biological safety laboratories.

Amino acid mutations in the HA protein are essential for highly efficient transmission in mammals [16], but mutations in other viral proteins are also necessary [14, 15]. Mutations in different proteins introduce synergy and nonlinearity among these viral proteins, which was supported by the results in the paper. The linear classifier (the KNN model) showed poor predictive performance on the initial set of 178 signature positions. Moreover, the minimal signature position set was composed by 63 amino acid residues and distributed among different viral proteins as shown in Table 2. This synergistic effect should be notable in further study. Moreover, the NP protein contained the largest number of signature positions (12 amino acid residues; about 12/63 = 19%), suggesting that NP is very important for host range of influenza virus [1]. The role of NP protein for transmission should be focused in the future.

The molecular characteristics of AA Factor 5 are related to electrostatic charge with high coefficients on isoelectric point and net charge [21]. Electrostatic charge is strong related with the binding of biology molecules, such as the binding between viral surface protein and host receptor, the binding between viral enzyme and host molecules. The poor performance of other four factors may suggest that host receptor binding, and viral polymerase activity play key roles for the adaption of human host and transmission of avian influenza virus with high efficiency.

Four popular classifiers were used to predict the phenotype of AIVs. With the empirical parameters, the SVM model achieved well performance while KNN not. The KNN parameters were adjust from k = 1 to 20 and the performance was still not good. The reason may be that the size of data was not adequate for the dimension of feature vector. In the paper, all of the 1783 influenza viruses in the final dataset were represented by a 178 × 5 = 890 dimension vector. The KNN algorism had weak performance for our data.

As shown in Table 3, three or four genome segments were needed for H7N9 and H9N2 virus to achieve the change of transmission phenotype with high probability (> = 0.90), which was very important for the surveillance of AIVs in the future. Moreover, when avian and human virus with the predicted genome pattern were founded in the same region or in the same case, the pandemic risk should be notable.

## Conclusions

The 178 signature mutations in 11 viral proteins were firstly screened by the random forest model. AIV pandemic risk was predicted by the SVM model with excellent performance. Although long evolution distance between avian and human influenza suggested that avian influenza virus in nature still need long time to fix among human, it should be notable that there are high pandemic risks for H7N9 and H9N2 AIVs. The novel findings in the paper provide important clues for pandemic surveillance.

## Methods

### Dataset

**Fig. 4:**
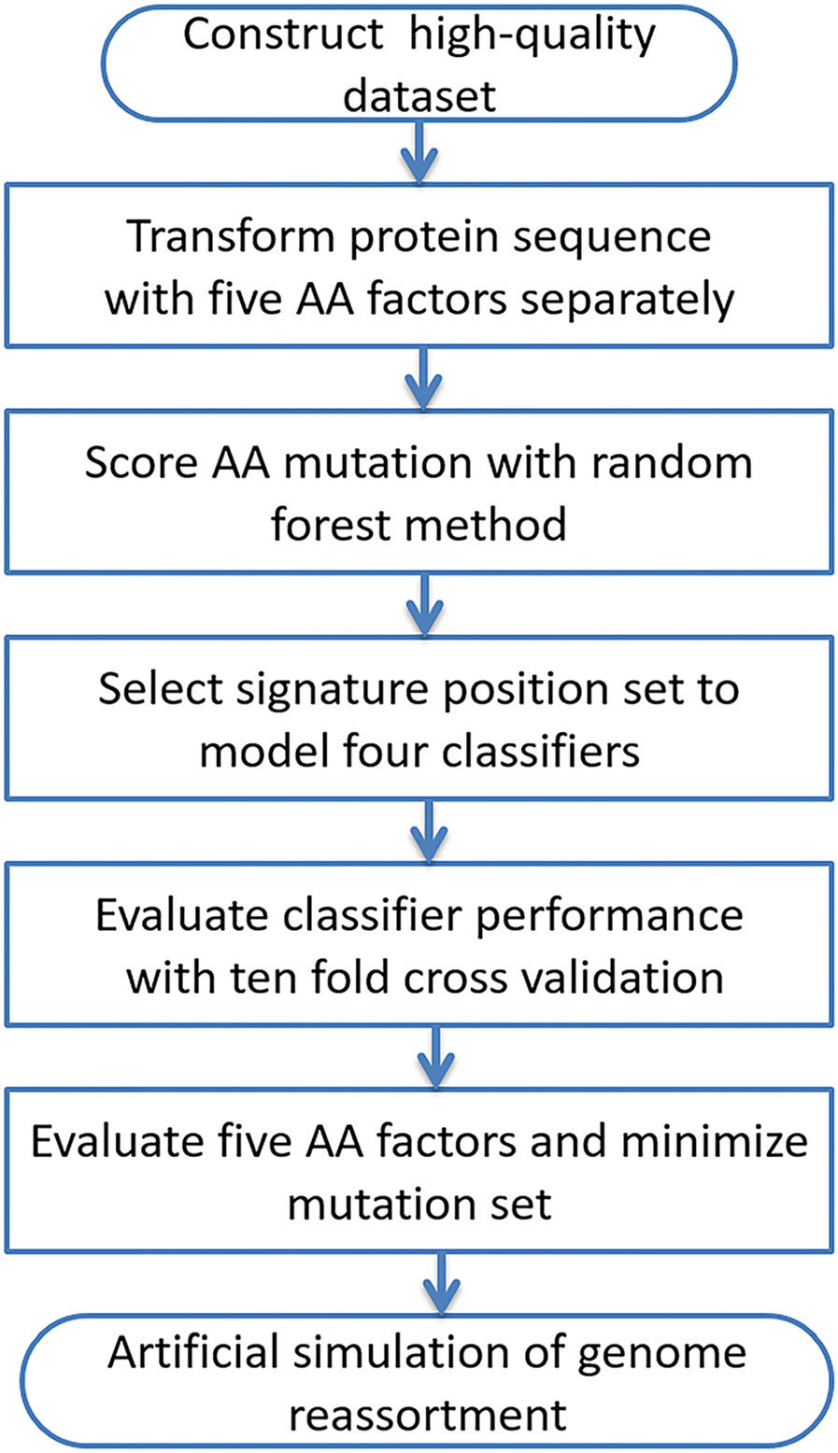
Flowchart of machine learning algorism used in the paper

The genome data of 16,551 influenza viruses isolated from nature were collected from the EpiFlu public database [30, 31] and those of six artificial H5N1 viruses with pandemic risk were collected from the ref. [14], which were processed and modeled using multiple public bioinformatics tools and algorithms as shown in Fig. 4. The strains were isolated between January 1996 and February 2016. The details for data cleaning are the same as those in the ref. [32–34].

The final dataset for predicting pandemic risk contained two category virus in the view of pandemic risk: 1) 869 high-quality AIV strains with low transmission efficiency among human: 440 avian-origin AIVs (H1–H14, H16 subtypes) and 429 human-origin AIVs (H5N1, H5N6, H7N3, H7N7, H7N9 and H9N2 subtypes); 2) 914 influenza strains with high transmission efficiency among human: 908 seasonal or pandemic human influenza (H1N1, H1N2 and H3N2 subtypes) and six artificial H5N1 viruses [14]. Considering the balance of data size and high similarity of viral protein sequence, seasonal and pandemic human virus in nature should differ by isolation location, isolation time, or antigen subtype. The information related to these strains is summarized in Additional file 1.

### Scoring amino acid mutation

Random Forest is a collection of a large number of decision trees. The contribution of each feature to each tree in the random forest were calculated. All of the features were ranked according to the average of contributions to all of the trees in the model. The random forest method is very popularly used for feature selection of prediction problems and can rank the importance of the features in a large scale to discriminate the different categories. In this paper, transmission phenotype of high efficiency was predicted to evaluate the pandemic risk. Before the construction of classifier models, molecular features associated with transmission efficiency were firstly screened. The positive samples (high transmission efficiency) and negative samples (low transmission efficiency) were then classified by their importance scores at each amino acid position.

The RF method was used to screen the signature mutation in the 11 viral proteins [35]. To facilitate the computing of importance scores, the 11 proteins in each strain were artificially concentrated as order: Polymerase basic protein 2 (PB2), Polymerase basic 1 (PB1), The second protein expressed in the PB1 gene (PB1-F2), Polymerase acidic protein (PA), Hemagglutinin (HA), Nucleoprotein (NP), Neuraminidase (NA), Matrix protein 1 (M1), Matrix protein 2 (M2), Non-structural protein 1 (NS1), Nuclear export protein (NEP). Numerical sequences of the amino acid factor were achieved with the transformation of the artificial protein with the length of 4620 amino acids. Any deletions or insertions in the protein were replaced by zeros. All of the viruses were processed sequentially and were input to the RF model for the ranking of signature position. Breiman’s random forest algorithm was used as default. As five factors were used to select the feature and construct the classifiers, the final importance score at each position was the sum of five calculations. In brief, highly scoring positions were important for distinguishing positive and negative samples. Signature positions with high scores were regarded as important amino acid mutations associated with the phenotype of highly efficient transmission.

### Constructing the predicting model

Two-class model was constructed to predict and evaluate the pandemic risk of AIVs in the paper. After the ranking of amino acid mutations in all of the 11 viral proteins, each strain was represented as a numeric vector of length 5 N, where N is the length of the screened amino acid residue set. The pandemic risk was then predicted by four popular machine learning models: 1) Support vector machine [36]. The optimal hyperplane is determined with the regularization parameter C (C = 1) and the radial basis function (RBF) as default. 2) Random forest [35]. The RF model was implemented with the default parameter in the package. 3) Naïve Bayes [36]. The NB model was also implemented with the default parameter in the package. 4) K-nearest neighbor [37]. The KNN classifier is a nonparametric method to determine a sample category by a majority vote of its neighbors; the number of neighbors in this paper was set to be 3 (k = 3). All of the four classifiers were implemented in the R environment and related packages.

### Evaluating the performance of different classifiers

All of the four models were trained on 823 positive samples (high transmission efficiency) and 782 negative samples (low transmission efficiency) randomly selected from the cleaned dataset of influenza virus. The remaining 10% of samples (91 positive and 87 negative samples) were reserved as an independent test dataset for assessing the performances of the classifiers. The 10-fold cross validation and the receiver operating characteristic curve were used to evaluate the performance of the SVM, NB, RF and KNN classifiers. The area under the ROC curve reveals the optimal parameters in the four classifiers. To compare the classifier performances, we repeated the evaluation process 100 times and plotted the distributions of the resulting AUC values. The AUC was calculated in R [38]. The AUC value ranges from 0 to 1. The performance and robustness of the four classifiers was evaluated by the AUC values and its distribution. The 1783 influenza viruses in the final dataset were shown by the multidimensional scaling method in R [37].

### Artificial simulation of genome reassortment

As human influenza virus and human-origin avian influenza virus existed simultaneously in nature, mix infection in one case could cause the occurrence of pandemic virus by the mechanism of genome reassortment [20]. The perfect SVM classifier was used to analysis the artificial stimulation of genome reassortments between three human-origin AIVs and three human viruses. The artificial data were treated and predicted as above. Platt scaling was used to transform the output of the SVM model into a probability over two classes and evaluated the pandemic risk of genome reassortment viruses.

In the paper, three human viruses with high efficiency of transmission in positive samples: A/Ohio/09/2015 (EPI_ISL_179403; H1N1), A/Wisconsin/13/2015 (EPI_ISL_176723; H3N2), and A/Sichuan/1/2009 (EPI_ISL_30411; H1N1; 2009 pandemic swine virus) and three human-origin avian viruses with low efficiency of transmission in negative samples: A/Egypt/682/2015 (EPI_ISL_195659; H5N1), A/Zhejiang/9/2015 (EPI_ISL_192505; H7N9) A/Hunan/44558/2015 (EPI_ISL_203644; H9N2) were used.

## Additional files

Additional file 1: The nomenclature for influenza virus in the final dataset. (XLSX 98 kb)

Additional file 2: The clustering details for the MDS method. (XLSX 135 kb)

## Abbreviations

AA: Amino acid
AIV: Avian influenza virus
AUC: Area under the ROC curve
HA: Hemagglutinin
KNN: K-nearest neighbor
M1: Matrix protein 1
M2: Matrix protein 2
NA: Neuraminidase
NB: Naïve bayes
NEP: Nuclear export protein
NP: Nucleoprotein
NS1: Non-structural protein 1
PA: Polymerase acidic protein
PB1: Polymerase basic 1
PB1-F2: The second protein expressed in the PB1 gene
PB2: Polymerase basic protein 2
RBF: Radial basis function
RF: Random forest
ROC: Receiver operating characteristic curve
SVM: Support vector machine
